# Discovery of molecular signature of long-term psychiatric sequelae in COVID-19 through proteome profiling of dried blood spots

**DOI:** 10.1038/s41398-025-03590-2

**Published:** 2025-10-10

**Authors:** Myungjae Baik, Jeonghun Yeom, Sang Min Lee, Hwangkyo Jeong, Ah Rah Lee, Seungyoon Seo, Sung Moon Choi, Yeonwoo Jo, Hye Yoon Park, Eun Young Kim, Jong-Woo Paik

**Affiliations:** 1 Department of Psychiatry, Kyung Hee University Hospital, Kyung Hee University School of Medicine, Seoul, Republic of Korea; 2 Prometabio Research Institute, Prometabio co., ltd. Hanam-si, Gyeonggi-do, Republic of Korea; 3 Postdoctoral Researcher, Industry-Academia Cooperation Foundation, Kyung Hee University, Seoul, Korea; 4 Department of Psychiatry, Seoul National University Hospital, Seoul National University College of Medicine, Seoul, Korea; 5 Department of Psychiatry, Seoul National University Health Service Center, Seoul, Republic of Korea; 6 Department of Human Systems Medicine, Seoul National University College of Medicine, Seoul, Republic of Korea

**Keywords:** Diagnostic markers, Depression, Pathogenesis

## Abstract

Neuropsychiatric sequelae represent a significant aspect of post-acute sequelae of SARS-CoV-2 (PASC, or long COVID), posing considerable public health challenges. This study identified molecular signatures associated with PASC in individuals with psychiatric morbidities via dried blood spot proteomic analysis. We evaluated 51 COVID-19 survivors ≥ 60 days post-infection, categorizing them into three groups: those with new-onset psychiatric disorders (n = 16, psychiatric PASC), those with persistent symptoms but no psychiatric disorders (n = 18, general PASC), and those symptomatically recovered (n = 17, recovered). Liquid chromatography-mass spectrometry analysis identified 1604 proteins. Differentially expressed proteins underwent Gene Ontology and Kyoto Encyclopedia of Genes and Genomes pathway enrichment analyses. Protein panels, including isoform 1 of fibronectin, sorbitol dehydrogenase, cytosolic acyl coenzyme A thioester hydrolase, and apolipoprotein A-II, differentiated psychiatric PASC from recovered individuals with an area under the curve (AUC) of 0.865 (95% CI: 0.658–1). Filamin A and vacuolar protein sorting-associated protein VTA1 homolog distinguished psychiatric PASC from general PASC at an AUC of 0.831 (95% CI: 0.6–1). Decision tree analysis revealed that alpha-synuclein, pyruvate kinase PKM, and sorbitol dehydrogenase effectively distinguished the three groups with 82% classification accuracy. These findings suggest that alterations in immune, glucose, and lipid metabolism pathways, along with neuroinflammation and neurodegeneration, contribute to the psychiatric PASC phenotype and highlight potential biomarkers for psychiatric disorders during the long-term COVID-19 clinical course.

## Introduction

An estimated 10–30% of COVID-19 survivors convalescing from severe acute respiratory syndrome coronavirus 2 (SARS-CoV-2) infection continue to experience post-acute sequelae of SARS-CoV-2 infection (PASC), commonly referred to as long COVID [1–3]. Long COVID is characterized by a constellation of lingering symptoms, including cognitive impairment, sleep disturbances, fatigue, dyspnea, and autonomic dysfunction, alongside numerous other manifestations [4]. These persistent symptoms represent an enormous burden of disease on both pulmonary and extrapulmonary systems [5], resulting in significantly decreased quality of life [6] and contributing to labor shortage [7]. Neuropsychiatric symptoms are a major long-term feature of long COVID [8–10]. A large multicenter study demonstrated that the prevalence of anxiety or depressive symptoms among patients discharged after COVID-19 was 24.5%, post-traumatic stress disorder (PTSD) was 12.2% [11], and cognitive impairment was 16.9%. A recent cohort study involving 236,379 participants reported substantial psychiatric morbidity (33.62%), encompassing mood disorders, anxiety, psychotic disorders, substance use disorders, insomnia, and dementia, 6 months following COVID-19 infection [12]. Another large-scale cohort study found a markedly elevated risk of new-onset neuropsychiatric disorders at 6 months post COVID-19 infection, including sleep-wake disorders, neurocognitive disorders, anxiety- and trauma-related disorders, alongside an increased burden of antidepressant and benzodiazepine, sedative, and anxiolytic agents [5]. Therefore, the long-term neuropsychiatric implications of COVID-19 present significant public health concerns. Understanding the precise mechanisms and establishing diagnostic certainty of neuropsychiatric sequelae is critical for ongoing monitoring and the development of effective interventions.

Research on the pathological mechanisms of neuropsychiatric PASC is still limited. Possible mechanisms include generalized neuroinflammation; microglial activation; and vascular damage due to coagulopathy, endothelial dysfunction, and neuronal injury [13]. In patients with PASC, previous studies have observed imbalances in neurotransmitters within the brain [14]. Furthermore, brain and brainstem hypometabolism correlates with insomnia and cognitive decline [15, 16], along with abnormal cerebrospinal fluid [17] and microglial reactivity akin to that observed in chemotherapy, known as “chemo-brain” [18]. Previous research indicates that neuropsychiatric symptoms in patients with PASC are related to a hyper-inflammatory state characterized by increased levels of pro-inflammatory cytokines such as interleukin (IL)-6, IL-2, IL-17, and tumor necrosis factor-alpha (TNF-α) [14, 19]. To date, few studies have clearly defined the molecular signatures of neuropsychiatric PASC, and research on potential molecular diagnoses in individuals exhibiting neurological symptoms remains limited. However, plasma proteome analyses in patients with PASC have demonstrated altered inflammatory and mitochondrial protein levels [20]. Proteomic and metabolomic and proteomic analyses of cerebrospinal fluid suggest that persistent mild neurological symptoms, such as headache, are associated with dysregulation of immune and metabolic pathways, including steroid hormone and fatty acid biosynthesis, and sphingolipid metabolism [17]. Nonetheless, proteomic analyses that delineate specific molecular features to determine and monitor PASC with persistent psychiatric symptoms have not been performed in detail.

In this study, dried blood spot (DBS) proteomic analysis was employed to identify molecular signatures capable of defining PASC with psychiatric morbidities compared to PASC without psychiatric disorders and recovered controls. Dried blood spots offer an innovative and minimally invasive sampling technique (e.g., remote or home self-sampling via finger prick), requiring minimal blood volumes and allowing convenient sample storage and easy shipment at room temperature for extended periods, all at low cost [21–24]. This approach is particularly advantageous for individuals quarantined because of infectious diseases or those with psychiatric disorders that pose recruitment challenges [25–27]. Signatures identified using DBS proteomics may serve as useful biomarkers for assessing and monitoring psychiatric disorders during the clinical course of individuals with SARS-CoV-2 infection.

## Materials and methods

### Participants and study design

This study included 51 participants from a post-acute sequelae of SARS-CoV-2 infection (PASC) clinic at Kyung Hee University Hospital between December 2022 and June 2023. The participants were divided into three groups: (1) 16 individuals with persistent symptoms lasting ≥60 days and clinically diagnosed with new-onset psychiatric disorders following acute, PCR-confirmed SARS-CoV-2 infection (psychiatric PASC); (2) 18 individuals with persistent symptoms lasting ≥60 days but without psychiatric disorders following an acute infection (general PASC); and (3) 17 individuals who symptomatically recovered after an acute infection (recovered). Psychiatric disorders were clinically diagnosed using the Mini-International Neuropsychiatric Interview (version 7) by a trained psychiatrist. “Recovered” participants had been diagnosed with COVID-19 at least 60 days before enrollment and had COVID-19-associated symptoms resolved at enrollment. The average time from the COVID-19 diagnosis to study registration and sample collection was 10.0 months. The exclusion criteria were the following: (1) systemic diseases (e.g., autoimmune or hematological diseases), (2) active stage of infections, (3) preexisting severe psychiatric disorders, (4) severe diseases of the central nervous system (CNS) (e.g., stroke), (5) history of malignant tumors, and (6) those who were pregnant, lactating, or menstruating. This study was conducted in strict adherence to the Declaration of Helsinki and relevant clinical research regulations. Approval was obtained from the Ethics Committee of Kyung Hee University Hospital (Approval Number: 2023-KY-004–03). Written informed consent was obtained from all participants, and strict adherence to the STROBE statement reporting guidelines was ensured.

Data were collected regarding previous and present medical histories, sociodemographic and lifestyle characteristics, COVID-19 treatment history, and symptom profiles. For comprehensive PASC symptom assessment, a list of 33 symptoms across nine categories, according to the National Institute for Health and Care Excellence (NICE) guidelines, was implemented [28]. Depressive symptoms and generalized anxiety were assessed using the Patient Health Questionnaire-9 [29, 30] and the Generalized Anxiety Disorder-7 scale [31, 32], respectively. Post-traumatic symptoms were assessed using the Primary Care PTSD Screen for DSM-5 [33, 34] and the Korean version of the Impact of Event Scale-Revised [35, 36]. The Fatigue Severity Scale [37] and Insomnia Severity Index [38] were utilized to assess fatigue and sleep disturbances. Quality of life was evaluated using the WHO Disability Assessment Schedule (WHODAS 2.0) 12-item version [39]. The degree of social support was assessed using the Duke-UNC Functional Social Support Questionnaire [40], where higher scores indicate less social support.

### DBS sample preparation

Whole blood (10 μL) was collected via finger prick into a Mitra® Clamshell (Neoteryx®, Torrance, CA, USA) and stored at room temperature. Each dried blood sample stored on the tip of the Mitra® device was removed and placed in a LoBind tube. Proteins were extracted in 500 μL of 5% sodium dodecyl sulfate and 50 mM triethylammonium bicarbonate buffer (pH 8.5) at 50 °C with vortexing at 1500 rpm for 10 min. The protein concentration of the collected supernatants was determined using a bicinchoninic acid protein assay kit (Thermo Fisher Scientific, Loughborough, UK). Proteins from the samples were digested into peptides using the S-Trap-based digestion platform, following the manufacturer’s protocol (PMID: 29754492). Briefly, the extracted DBS samples (80 μg protein) were reduced and alkylated using tris(2-carboxyethyl) phosphine hydrochloride (Thermo Fisher Scientific, Waltham, MA, USA) and iodoacetamide (Sigma-Aldrich, St. Louis, MO, USA). Phosphoric acid (Sigma-Aldrich) was added to the samples to achieve a final concentration of 1.2%. Aqueous methanol (90%) with 100 mM TEAB was added to the acidified sample and loaded onto an S-Trap microcolumn. The bound proteins were washed three times, and a trypsin/Lys-C mixture (Promega, Madison, WI, USA) in 50 mM TEAB at a protein ratio of 25:1 was added directly to the column. After incubation, the peptides were eluted in three steps. The peptide-eluted samples were dried using a speed vacuum concentrator with a cold trap (CentriVap Cold Traps; Labconco, Kansas City, MO, USA).

### Liquid chromatography (LC)-mass spectrometry (MS)

The dried peptide samples were reconstituted with 0.1% formic acid. The total peptide concentration was measured using a UV/Vis spectrophotometer at a wavelength of 280 nm. Each sample was dissolved at a concentration of 0.5 μg/μL. Samples (1 μL) were injected and analyzed employing a SCIEX ZenoTOF 7600 mass spectrometer system (AB SCIEX, Framingham, MA, USA). For LC separation, the Vanquish Neo UHPLC system (Thermo Fisher Scientific, Waltham, MA, USA) was used with a PepMapTM Neo trap cartridge (300 μm × 5 mm, 5 μm, 120 Å) as the trap column, and a Phenomenex column (Kinetex XB-C18, 150 × 0.3 mm I.D., 3 μm, 100 Å) as the analytical column. The column temperature was maintained at 40 °C. The samples were separated for 35 min using solvents A (0.1% formic acid) and B (0.1% formic acid in 100% acetonitrile). Solvent B was supplied at a flow rate of 5 μL/min and increased from 3–25% over 25 min, from 25–32% over 2 min, and from 32–80% over 1 min, maintained at 80% for 2 min, reduced to 3% over 1 min, and maintained at 3% for an additional 4 min. The samples were acquired using Zeno sequential window acquisition of all theoretical mass spectra (SWATH) data collection: a data-independent acquisition (DIA) technique that uses Zeno trap pulsing—a linear ion trap pulse—to increase sensitivity over the existing SWATH method (PMID: 36449390). The Zeno SWATH parameters were as follows: lower m/z limit, 400; upper m/z limit, 900; window overlap (Da), 1.0; collision energy (CE), 10; declustering potential, 80. MS2 spectra were collected in the 100–1500 m/z range, with an accumulation time of 20 ms. Other MS parameters were set as follows: ion source gas 1 (GS1), 12; ion source gas 2 (GS2), 60; curtain gas (CUR), 25; CAD gas, 7; temperature (TEM), 250 °C; ion spray (IS), 4500.

### SWATH data analysis

The SWATH files for each sample were processed in library-free mode using DIA-NN (version 1.8.1) [41]. This approach facilitated the analysis of the FASTA database (Human SwissProt, April 2022) alongside a pan-human spectral library [42]. The search parameters for DIA-NN were set as follows: precursor false discovery rate (FDR) = 1%, isotopologs turned on, match-between-runs (MBR) turned on, protein inference at the gene level, quantification strategy set to Robust LC (high precision), neural network classifier double-pass mode, and cross-run normalization set to retention time (RT)-dependent. Protein re-annotation was performed. Precursor ion generation included the following parameters: trypsin/P with a maximum of one missed cleavage; protein N-terminal M excision enabled; carbamidomethyl on C as a fixed modification; peptide length from 7–30; precursor charge range of 1–4; precursor m/z from 400–900; and fragment ion m/z from 100–1500. Protein quantification results were further processed and analyzed using R (version 4.2.1) with the diann-r package (https://github.com/vdemichev/diann-rpackage). Precursor and protein-group FDR were filtered through a 1% threshold. Genes were identified and quantified using proteotypic peptides. The protein groups were quantified using the MaxLFQ algorithm. Statistical analysis of each dataset was performed using Perseus (version 1.6.15.0) and MetaboAnalyst 6.0 (https://www.metaboanalyst.ca). Gene enrichment analysis was performed using Shiny Gene Ontology (GO) analysis (http://bioinformatics.sdstate.edu/go/).

## Results

### Demographic and clinical characteristics

The demographic characteristics of the study cohort are shown in Table 1. The cohort consisted of 16 adults (all women, mean (SD) age, 44.6 (16.1) years) with general PASC symptoms and diagnosed with psychiatric disorders (“Psychiatric PASC”), 18 adults (all women; mean (SD) age, 46.3 (8.5) years) with general PASC symptoms but without psychiatric disorder (“General PASC”), and 17 adults (1 men, 16 women; mean (SD) age, 42.8 (11.8) years) who symptomatically recovered (“Recovered”). Most participants had mild COVID-19 symptoms during acute infection. Most patients receive symptomatic treatment while quarantined at home. Only two participants were hospitalized, and no participant required ICU care for mechanical ventilation. Compared to other groups, the participants in the psychiatric PASC group had lower levels of education and reported a decrease in income compared to before infection. There were no significant differences among the groups in terms of physical comorbidities or psychiatric treatment history. The mean (SD) total number of PASC symptoms (range, 0–33) was 10.4 (6.2) in the psychiatric PASC and 8.5 (5.4) in the general PASC, and 1.1 (1.5) in the recovered group. Participants in the psychiatric PASC group had significantly higher levels of depressive symptoms, anxiety, and post-traumatic stress symptoms and lower levels of functional social support than those in the other groups. Individuals in the psychiatric and general PASC groups reported elevated levels of sleep disturbance and fatigue and lower quality of life than those in the recovered group.

**Table 1:** Demographic and clinical characteristics of participants with long COVID symptoms.

	Psychiatric PASC (n = 16)	General PASC (n = 18)	Recovered (n = 17)	*p*-value
Age, years (SD)	44.6 (16.1)	46.3 (8.5)	42.8 (11.8)	0.696
Female, n (%)	16 (100.0)	18 (100.0)	16 (94.1)	0.647
Education years, n (%)				
0-6	0 (0.0)	0 (0.0)	1 (5.9)	0.003
7-12	10 (66.7)	2 (11.1)	4 (23.5)	
≥13	5 (33.3)	16 (88.9)	12 (70.6)	
Change of income level				
Decreased	11 (68.8)	7 (38.9)	3 (17.6)	0.008
No change	4 (25.0)	10 (55.6)	14 (82.4)	
Increased	1 (6.3)	1 (5.6)	0 (0.0)	
History of psychiatric disease, yes, n (%)	2 (12.5)	3 (16.7)	0 (0.0)	0.167
Comorbid physical illness, yes, n (%)	7 (43.8)	3 (16.7)	3 (17.6)	0.169
Stroke	0 (0.0)	0 (0.0)	0 (0.0)	-
Hypertension	3 (18.8)	1 (5.6)	2 (11.8)	0.415
Cardiovascular	1 (6.3)	0 (0.0)	0 (0.0)	0.314
Diabetes	0 (0.0)	1 (5.6)	1 (5.9)	1.000
Hyperlipidemia	5 (31.3)	2 (11.1)	1 (5.9)	0.139
Pulmonary disease	1 (6.3)	0 (0.0)	0 (0.0)	0.314
Hepatic disease	0 (0.0)	0 (0.0)	0 (0.0)	-
Others (cancer, etc)	2 (12.5)	4 (22.2)	0 (0.0)	0.124
Vaccinated, yes, n (%)	15 (93.8)	18 (100.0)	16 (94.1)	0.534
Treatment history of COVID-19				1.000
ICU care	0 (0.0)	0 (0.0)	0 (0.0)	
Hopital admission	1 (6.3)	1 (5.6)	0 (0.0)	
COVID-19 residential treatment centers	0 (0.0)	0 (0.0)	1 (5.9)	
Home quarantine and treatment	15 (93.8)	16 (88.9)	16 (94.1)	
No specific treatment	0 (0.0)	1 (5.6)	0 (0.0)	
Alcohol drinking				0.672
None	6 (37.5)	5 (27.8)	4 (23.5)	
<1/week	7 (43.8)	6 (33.3)	6 (35.3)	
≥2/week	3 (18.8)	7 (38.9)	7 (41.2)	
Smoking, n(%)				0.414
Never	15 (93.8)	18 (100.0)	16 (94.1)	
Past	0 (0.0)	0 (0.0)	1 (5.9)	
Current	1 (6.3)	0 (0.0)	0 (0.0)	
Long COVID symptoms, total score (SD)	10.4 (6.2)	8.5 (5.4)	1.1 (1.5)	<0.001
Respiratory symptom	0.8 (0.6)	0.6 (0.6)	0.1 (0.3)	0.002
Cardiovascular symptom	0.8 (1.0)	0.6 (0.8)	0.1 (0.5)	0.066
Generalized symptom	1.3 (0.7)	1.4 (0.6)	0.4 (0.5)	<0.001
Neurological symptom	2.1 (1.8)	2.1 (1.6)	0.2 (0.4)	<0.001
Gastrointestinal symptom	0.9 (1.1)	0.5 (0.6)	0.1 (0.2)	0.012
Musculoskeletal symptom	0.8 (0.8)	0.8 (0.9)	0.1 (0.2)	0.003
Ear, nose, throat symptom	2.1 (1.7)	1.4 (1.7)	0.2 (0.4)	0.001
Dermatological symptom	0.6 (0.6)	0.5 (0.6)	0.0 (0.0)	0.004
Psychiatric symptom	1.3 (1.0)	0.5 (0.7)	0.0 (0.0)	<0.001
Patient Health Questionnaire-9, total score (SD)	11.2 (5.8)	6.3 (6.0)	1.3 (2.7)	<0.001
Generalized anxiety disorder-7, total score (SD)	7.4 (4.9)	2.0 (2.1)	0.8 (2.2)	<0.001
Primary Care PTSD Screen, total score (SD)	2.1 (1.8)	0.8 (1.3)	0.5 (0.9)	0.004
Impact of Event Scale-Revised, total score (SD)	62.4 (31.2)	41.0 (18.0)	26.4 (10.5)	<0.001
Fatigue severity scale, total score (SD)	42.9 (11.2)	42.1 (9.3)	26.1 (11.1)	<0.001
Insomnia Severity Index, total score (SD)	17.9 (7.3)	17.2 (7.9)	12.5 (4.0)	0.043
WHO Disability Assessment Schedule, total score (SD)	24.5 (8.0)	21.3 (10.7)	13.7 (2.6)	0.001
Functional social support questionnaire, total score (SD)	26.7 (10.3)	16.2 (7.1)	12.2 (3.8)	0.008

### Proteomic analysis of DBS samples using LC-MS/MS

**Fig. 1:**
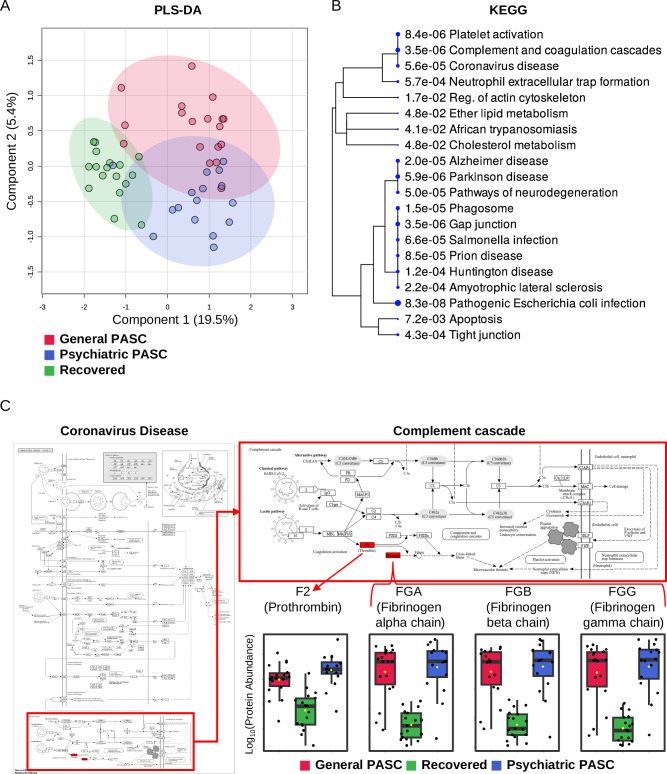
Features of dried blood proteome from patients with COVID-19. **A** PLS-DA analysis results of dried blood proteome in 51 PASC samples; **B** KEGG pathway for 17 proteins with significant differences through ANOVA statistical analysis between the three groups corresponding to psychiatric PASC, general PASC, and recovered. The number express P-value, and the size of the circle represents the number of genes included in the pathway. **C** Location of proteins involved in the Coronavirus disease pathway among 17 significant proteins and expression patterns of proteins by group.

To identify the molecules that define psychiatric PASC, we performed proteomic analyses of DBS samples obtained from 51 patients. We introduced the SWATH-MS technique [43], which enhances the reproducibility of quantified proteins, and performed multivariate analysis to characterize the protein signatures of these DBS samples. LC-MS/MS quantified a total of 1604 proteins. The median sample coefficient of variation was 19% for both the psychiatric and general PASC groups and 20% for the recovered group, indicating that abundant peptides or proteins were identified and quantified by MS without bias. Partial least squares-discriminant analysis (PLS-DA) based on quantitative proteomic changes demonstrated the separation of psychiatric PASC, general PASC, and recovered groups into two components: component 1,19.5%; component 2,5.4% (Fig. 1A). Statistical analysis using one-way ANOVA identified 17 proteins that differed significantly among the three groups (Supplementary Table 1). To annotate the potential functions of these 17 significant proteins, Kyoto Encyclopedia of Genes and Genomes (KEGG) pathway and Gene Ontology analyses were conducted using the Shiny GO web tool (http://bioinformatics.sdstate.edu/go/)(PMID: 31882993), which revealed associations with “Coronavirus disease” (Fig. 1B). Four proteins––prothrombin, fibrinogen alpha chain, fibrinogen beta chain, and fibrinogen gamma chain––were found at higher concentrations in the psychiatric and general PASC groups than in the recovery group (Fig. 1C).

### Identifying biomarkers

**Fig. 2:**
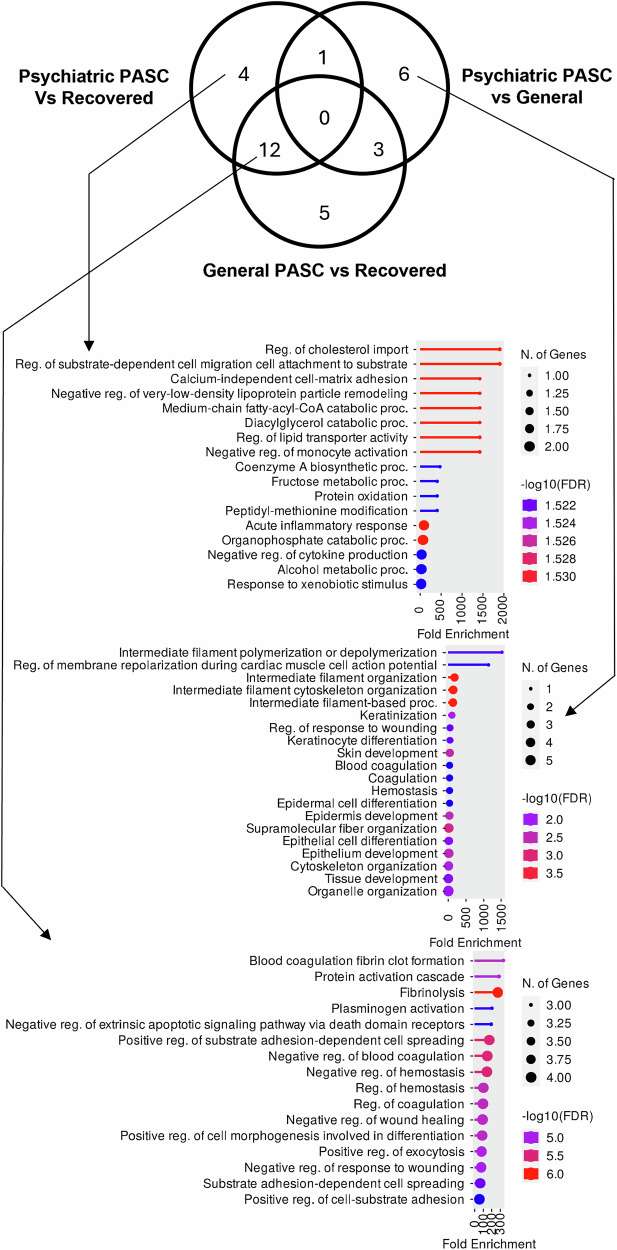
Biological process in psychiatric PASC-associated DEPs. Venn diagram for DEPs identified through individual pairwise comparisons and biological processes from gene enrichment analysis for psychiatric PASC-specific DEPs.

Differentially expressed proteins (DEPs) were identified using Student’s t-test (P value ≤ 0.05, ratio ≥ 1.3 or ≤ 0.8) across three comparisons: recovered vs. psychiatric PASC, recovered vs. general PASC, and general vs. psychiatric PASC (Supplementary Table 2). Venn diagram analysis of the identified DEPs was conducted, followed by GO analysis to determine the biological processes involved (Fig. 2). The four DEPs identified exclusively in the recovered vs. psychiatric PASC comparison were related to processes such as calcium-independent cell-matrix adhesion, regulation of substrate-dependent cell migration to the cell substrate, and regulation of cholesterol import. In the general vs. psychiatric PASC comparison, the six DEPs identified were related to intermediate filament polymerization or depolymerization, and regulation of membrane repolarization during cardiac muscle cell action potential. The 12 DEPs found in both the recovered vs. psychiatric PASC and recovered vs. general PASC comparisons were involved in protein activation cascade, blood coagulation fibrin clot formation, and fibrinolysis. Lastly, five DEPs identified exclusively in the recovered versus general PASC comparison did not exhibit any specific biological processes (Fig. 2).

**Fig. 3:**
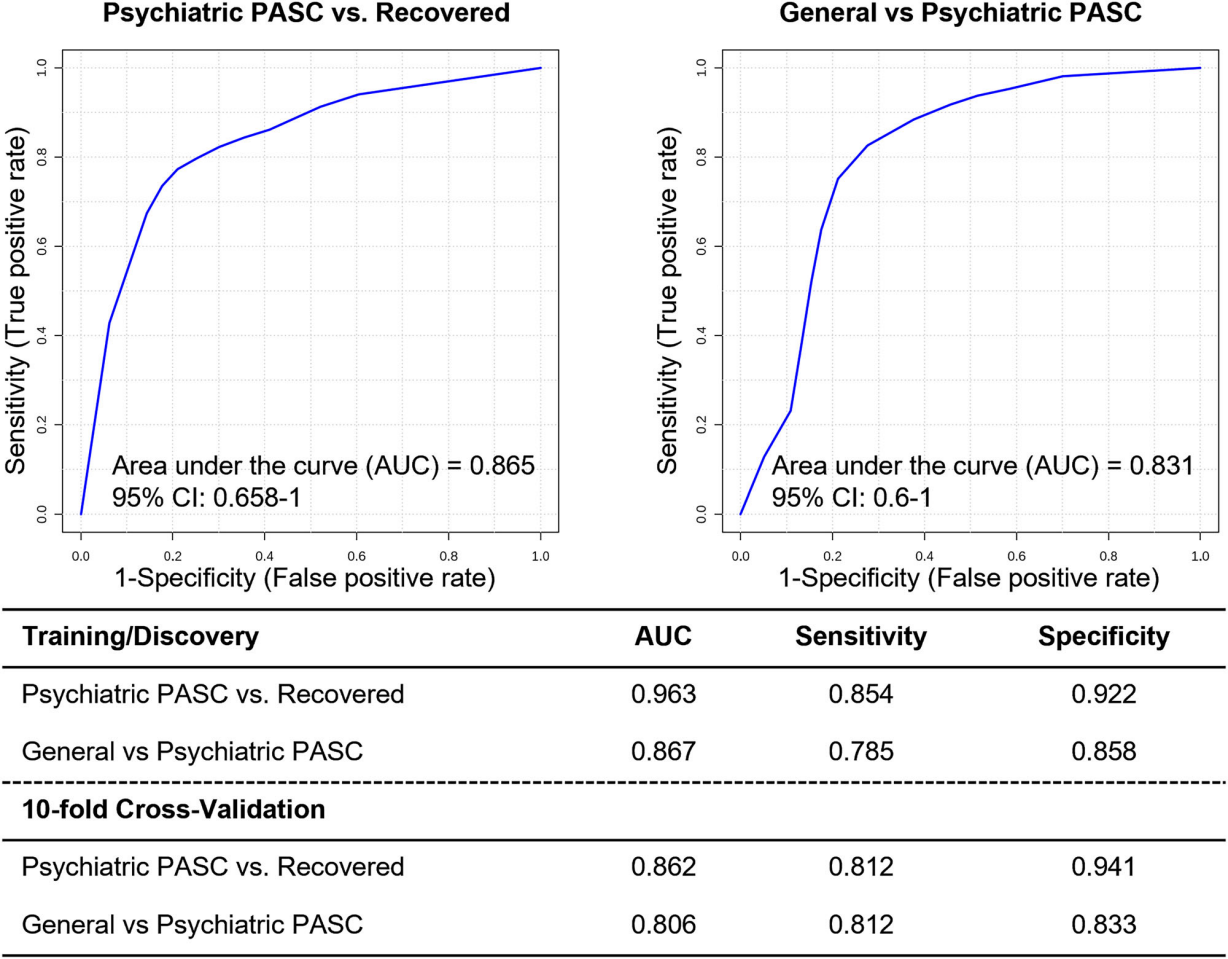
ROC curve for proteins selected through each pairwise comparison. The upper panel was created using the logistic regression method for four proteins that had significant differences only in the psychiatric PASC group compared to the recovered group. The bottom panel is the same as the analysis above for two proteins that showed significant differences only in the psychiatric PASC group compared to the general PASC group.

A multiple protein panel analysis based on the logistic regression method was performed using the four DEPs (isoform 1 of fibronectin, sorbitol dehydrogenase, cytosolic acyl coenzyme A thioester hydrolase, and apolipoprotein A-II) identified exclusively in the comparison between the recovered and psychiatric PASC groups, resulting in an area under the curve (AUC) of 0.865 (95% CI: 0.658–1) (Fig. 3). Similarly, multiple protein panel analyses using two of the six DEPs (filamin-A and vacuolar protein sorting-associated protein VTA1 homolog) identified exclusively in the comparison between the general and psychiatric PASC groups (excluding keratin and immunoglobulin) yielded an AUC of 0.831 (95% CI: 0.6–1).

**Fig. 4:**
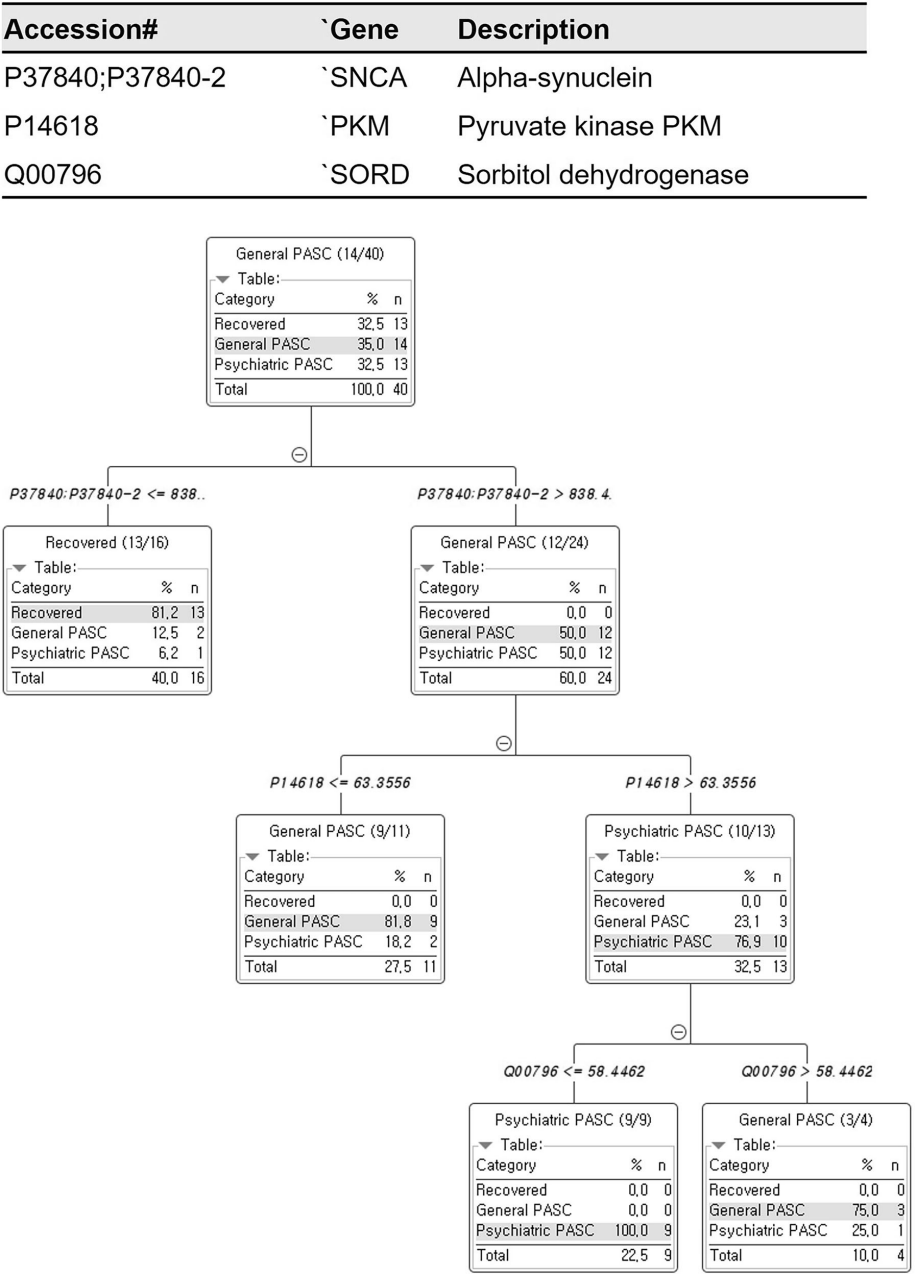
Classification of patients with PASC based on protein level in DBS. Three proteins were used in the decision tree; the protein used in the first prediction model was alpha-synuclein, which was divided into recovery and symptom groups based on the protein standardized intensity value of 838.4. The protein used in the second prediction model was pyruvate kinase PKM, classified into psychiatric PASC and general PASC groups based on the value of 6.3556. The last model classified the symptom group based on the sorbitol dehydrogenase value of 58.4462.

Furthermore, we aimed to identify biomarkers by implementing a decision model, an interpretable approach that directly predicts and classifies three types of disease states (Fig. 4). We began by selecting 607 proteins that were consistently and reproducibly identified across all samples from a total of 1604 detected proteins. Next, we divided our dataset into a training set consisting of 40 individuals (80% of the total) and a test set of 11 samples, utilizing the KNIME Analytics Platform [44]. Each node in the workflow was executed with default settings. The algorithm automatically selected discriminative proteins from the list of 607 and generated a decision tree with three key nodes and corresponding quantitative thresholds. This analysis revealed that alpha-synuclein was significantly associated with recovered and general PASC classifications, while pyruvate kinase PKM and sorbitol dehydrogenase were significantly associated with general and psychiatric PASC classifications. Alpha-synuclein was increased in general and psychiatric PASC compared to recovered PASCs, and pyruvate kinase PKM was most highly expressed in psychiatric PASC, followed by recovered and general PASC. Finally, sorbitol dehydrogenase was similarly expressed in general and recovered PASC, but it was less expressed in psychiatric PASC than in general and recovered PASC (Supplementary Fig. 1).

## Discussion

In this study, we investigated a DBS proteomic signature that identifies individuals with PASC who have been diagnosed with psychiatric disorders 8 weeks after COVID-19 infection using DBS. Protein panels, including isoform 1 of fibronectin, sorbitol dehydrogenase, cytosolic acyl coenzyme A thioester hydrolase, and apolipoprotein A-II, differentiated psychiatric PASC from the recovered group. Filamin A and vacuolar protein sorting-associated protein VTA1 homolog distinguished psychiatric PASC from general PASC. These proteins, which are involved in the cytoskeleton, cell membranes, and immune response, have shown potential as biomarkers of COVID-19 infection in recent literature. Decision tree analysis illustrated that three proteins––alpha-synuclein, pyruvate kinase PKM, and SORD––performed well in distinguishing the three groups. These proteins indicate that dysregulation in immune, glucose, and lipid metabolism pathways triggered by SARS-CoV-2 infection, coupled with ensuing neuroinflammation and neurodegeneration, contribute to the pathophysiology of psychiatric PASC. Our findings elucidate potential molecular mechanisms underpinning psychiatric PASC and propose valuable biomarkers for identifying psychiatric disorders throughout the prolonged clinical trajectory of COVID-19.

Multiple protein panels, including sorbitol dehydrogenase, cytosolic acyl coenzyme A thioester hydrolase, apolipoprotein A-II, and isoform 1 of fibronectin, could distinguish PASC with psychiatric disorders from recovered controls, with an AUC of 0.865 (95% CI: 0.658–1). Sorbitol dehydrogenase is the enzyme in the polyol pathway of glucose metabolism that converts sorbitol to fructose [42]. Sorbitol dehydrogenase deficiency in tissues leads to sorbitol accumulation under conditions of hyperglycemia and the subsequent amelioration of tissue function, such as degenerative neuropathy in diabetes [42]. Cytosolic acyl coenzyme A thioester hydrolase (also known as acyl-CoA thioesterase 7) is largely expressed in the brain [43], especially in oligodendrocytes and neurons. This protein could protect nerve cells from toxic accumulation of long-chain fatty acids and neuroinflammation [44, 45]. Furthermore, one recent study suggests that cytosolic acyl coenzyme A thioester hydrolase can modulate the amyloidogenic pathway of amyloid precursor protein metabolism and exhibits superior efficacy as a serum candidate biomarker of Alzheimer’s disease with a diagnostic accuracy of 77% compared with the conventional marker Aβ_42/40_ ratio [46].

Apolipoprotein-II is abundant in high-density lipoprotein (HDL); it plays a key role in lipid metabolism and is associated with cardiovascular diseases and metabolic syndromes [47]. Alterations in lipid metabolism manifest clearly in most patients with PASC [48]. In patients with COVID-19, a decrease in serum cholesterol levels has been reported [49]. In particular, COVID-19 severity was positively correlated with a decrease in HDL-cholesterol levels [50]; levels of the apolipoproteins related to HDL, such as apoA-I and apoA-II, were also decreased in COVID-19 [51]. A recent study showed lower levels of apoA-II in patients with Alzheimer’s disease or mild cognitive impairment compared to those in individuals with normal cognitive function [52]. Apolipoprotein-II has also been reported to be associated with antidepressant responses [53] and severity of fatigue in patients with multiple sclerosis [54]. Overall, the proteomic signatures of psychiatric PASC exclusively distinguished from recovered individuals revealed that neurodegeneration and neuroinflammatory processes in response to COVID-19 infection are closely associated with persistent psychiatric morbidity.

Fibronectin is an extracellular matrix protein that plays a key role in several biological processes, including thrombosis, hemostasis, infection, inflammation, and wound healing [55, 56]. A recent study reported that loss-of-function genetic variants in fibronectin 1 may reduce APOEε4-related Alzheimer’s disease risk [41]. On the other hand, the extracellular-matrix protein fibronectin-1 has shown to be associated with the variable “year ended full-time education” (p < 0.05; Proteome-Phenome Atlas) in a large-scale plasma-proteomics study of approximately 50,000 middle-aged individuals [57]. In our cohort, participants in the psychiatric PASC group had lower education levels compared to other groups, suggesting that education may be a potential confounding factor. However, after excluding the education-related FN1 and re-analyzing the data with only three significantly differentially expressed proteins (sorbitol dehydrogenase, cytosolic acyl-coenzyme A thioester hydrolase, and apolipoprotein A-II), the receiver-operating characteristic area under the curve improved from 0.865–0.897 (data not shown). This indicates that the proposed biomarker panel is largely independent of educational level as a confounder. Nonetheless, the interactions between socioeconomic variables and the proteome are too complex to be captured by a single indicator like education. Heterogeneity in income, job security, neighborhood environment, and health behaviors within the same educational stratum can still diversify protein expression profiles. Therefore, future studies should incorporate multidimensional socioeconomic variables and lifestyle data, applying longitudinal, multi-omics integration to delineate mediating and moderating pathways with greater precision.

Likewise, a multiple protein panel analysis using two of the six DEPs (filamin-A and vacuolar protein sorting-associated protein VTA1 homolog) distinguished psychiatric PASC groups from general PASC groups, with an AUC of 0.823 (95% CI: 0.585–1). These proteins have recently emerged as important biomarkers of COVID-19 and related morbidities. Filamin A is a large actin-binding cytoskeletal protein. It is crucial for cell motility as it stabilizes and integrates actin networks into cell membranes. Filamin A regulates the functions of both macrophages and endothelial cells, which are important for the progression of atherosclerosis and myocardial infarction, respectively [58]. Generalized cerebrovascular dysfunction, such as thrombotic microangiopathy, is associated with neurological manifestations of COVID-19 [13]. A recent proteomics study suggested that filamin A can be considered a potential biomarker for cardiac failure and myocardial infarction in patients with COVID-19 [59].

Moreover, filamin-A in extracellular vesicles represents a novel blood biomarker for human myalgic encephalomyelitis/chronic fatigue syndrome (ME/CFS) [60], which has been reported as a significant overlapping symptom in patients with long COVID [61]. PASC and ME/CFS share multiple biological abnormalities, given that both conditions are persistent neuroinflammatory and neuroimmune illnesses that are most commonly triggered by infection [62]. Filamin A can distinguish ME/CFS from idiopathic chronic fatigue and clinical depression [60], which is consistent with our findings. The vacuolar protein sorting-associated protein VTA1 is a component of the endosomal sorting complexes required for transport (ESCRT), which sorts membrane proteins such as lysosomal enzymes, lipids, and stimulated growth factor receptors, for degradation in lysosomes [63]. The ESCRT pathway plays critical roles in cellular processes such as cell surface growth factor receptor regulation [57, 64], regulation of HIV infection [65], and cytokinesis [66, 67]. Furthermore, a recent integrated multi-omics analysis suggested that the ESCRT pathway might play a key role in mediating SARS-CoV-2 infection, and VTA1 was identified as a critical host anti-viral factor [68]. VTA1 is also related to the biological pathways of cell cycle regulation, DNA damage response, and epithelial adherent junction signaling and may play important roles in cell death regulation and epithelium integrity by stress and viral infections [68].

To design a protein panel with clinical utility for simultaneously discriminating the three groups of this study, including the recovered group, we constructed a decision tree by dividing the participant sample into a small-scale training set and a test set, owing to limitations in sample collection. Alpha-synuclein, pyruvate kinase PKM, and sorbitol dehydrogenase distinguished the three groups with 82% accuracy. First, alpha-synuclein (α-Syn) was significantly increased in both psychiatric and general PASC groups; it is mainly expressed in nerve cells both in the central and peripheral nervous system, as well as in most immune cells and erythrocytes, which are key proteins in neurodegenerative disorders such as Parkinson’s disease [69, 70]. Moreover, α-Syn has been considered as a native antiviral factor within nerve cells [71, 72]. Most recently, abnormal SARS-CoV-2 nucleocapsid protein has been shown to accelerate alpha-synuclein aggregation and induce a Lewy body-like pathology (in vitro) [73]. This is consistent with SARS-CoV-2 infection triggering neuroinflammation and facilitating subsequent neurodegeneration, leading to neuropsychiatric symptoms in patients with PASC [74].

Additionally, pyruvate kinase PKM and sorbitol dehydrogenase were identified to distinguish psychiatric PASC from general PASC. Both proteins are involved in glucose metabolism-related neurodegeneration. pyruvate kinase PKM is a critical rate-limiting enzyme in the last step of glycolysis, in which phosphoenolpyruvate is converted to pyruvate and contributes to metabolic reprogramming. Metabolic reprogramming, observed in neurodegenerative diseases, viral infection, and cancer [75–77], refers to cellular metabolism alteration to support the increasing energy demand. Previous studies have shown that SARS-CoV-2 reprograms metabolism to support viral replication [78, 79]. pyruvate kinase PKM has also been related to several neuropsychiatric diseases, such as Alzheimer’s disease [80], Tourette syndrome, obsessive-compulsive disorder [81], and schizophrenia [82], as a reflection of glucose metabolism dysregulation. Moreover, plasma pyruvate levels were increased in the metabolomic analysis of patients with long COVID, which might be a result of both protein degradation and glycolytic dysregulation [83]. Furthermore, sorbitol dehydrogenase demonstrated the ability to identify psychiatric PASC in fully recovered COVID patients in the AUC analysis and is involved in degenerative neuropathy related to dysfunctional glucose metabolic pathways. A previous study suggested that PASC is related to new-onset insulin resistance, which might result in incident depressive symptoms by elevating overall neurotoxicity [84].

The DBS used in this study is a patient-centered sampling technique that has significant advantages in that it is easy to collect and store, requires a small amount of blood, and is widely used in clinical newborn screening [85]. Recently, various studies have attempted to analyze various substances, such as nucleic acids, heavy metals, and proteins, in addition to the main substances analyzed by DBS, such as chemicals and metabolites [86–88]. Although the research targeting these proteins is still in its infancy, our study was able to identify disease characteristics through proteomes extracted from DBS and discovered meaningful protein biomarkers for diagnosis. Our results suggest that DBS is an easy, fast, and cost-effective strategy for monitoring short- and long-term outcomes.

However, this study has some limitations. Given that PASC symptoms pose a high degree of inter-individual variability, the classification of clinical phenotypes into psychiatric and general PASC can be considered somewhat arbitrary. Moreover, it is difficult to ascertain which symptoms might be a consequence of COVID-19 infection—coincidental or as an aggravation of preexisting conditions. However, few studies have systematically classified the clinical phenotypes of PASC. Of the 3762 participants in an online study of people with persistent symptoms after COVID-19 infection, most participants reported persistent symptoms for up to 7 months, including prominent neuropsychiatric symptoms [89]. Another metabolomic study classified patients with PASC as class A (less than five symptoms, mainly neuropsychiatric disorders) and class B (five or more symptoms, with a broad spectrum of systemic disorders). Evidently, there is a specific population that predominantly exhibits neuropsychiatric symptoms during the PASC phase. At this stage, classification based on clinical diagnoses of psychiatric disorders, as in our study, is more appropriate than symptom-based approaches (e.g., counting psychiatric symptoms or focusing on specific symptoms like depressive mood) for identifying groups requiring early screening and intervention. One limitation of this study is its relatively small and homogeneous sample, which consisted predominantly of female participants. Consequently, the findings may not be fully generalizable to more diverse populations, including males and individuals from various cultural and demographic backgrounds. Additionally, potential confounders, such as baseline differences in demographics (e.g., education), lifestyle factors, and preexisting psychiatric history among participants, may influence the results. Future research should aim to replicate these findings in larger and more diverse cohorts while incorporating advanced statistical controls to enhance the external validity and applicability of the results across different groups.

The enormous disease burden attributable to psychiatric PASC worldwide [89] and heterogeneity among individuals with PASC require serious attention in terms of both accurate diagnosis and potential therapeutic intervention. Using DBS proteomics, we identified protein signatures that can be employed as multiple-protein panels for psychiatric PASC with a high degree of precision. Overall, these proteins suggest that alterations in the immune, glucose, and lipid metabolism pathways in response to SARS-CoV-2 infection and subsequent neuroinflammatory and neurodegenerative changes are involved in the clinical phenotype of PASC, specifically related to psychiatric morbidity. The findings of this study provide valuable insights that can inform future intervention strategies for long COVID. In particular, the application of DBS proteomics in clinical settings holds promise for enhancing early detection through specific biomarkers, personalized treatment approaches, and cost-effective monitoring of disease progression. DBS proteomics offers a minimally invasive and accessible method for biomarker analysis, making it especially beneficial for patients who face challenges in visiting clinics, such as those with COVID-19, psychiatric disorders, or other conditions requiring quarantine. Additionally, DBS is particularly well-suited for screening long COVID symptoms in individuals with a history of COVID-19 infection, as it enables remote and repeated monitoring of relevant biomarkers without the need for direct clinical visits. Future research should focus on validating these findings in larger cohorts and exploring how DBS-based biomarker profiling can be integrated into routine clinical practice to optimize patient care and outcomes.

## Supplementary information

supplementary material legends

expression level of three proteins

List of processed proteins derived from DBS

## Data Availability

The datasets generated and analyzed during the current study are not publicly available due to privacy and ethical restrictions but are available from the corresponding author on reasonable request.
