# Supplementary material for: Discovery of molecular signature of long-term psychiatric sequelae in COVID-19 through proteome profiling of dried blood spots
Source: Transl Psychiatry. 2025 Oct 10;15:389. doi: 10.1038/s41398-025-03590-2 (PMC12514250; doi:10.1038/s41398-025-03590-2)
Supplement: Supplementary file 1 — supplementary material legends [file 41398_2025_3590_MOESM1_ESM.docx]

**Supplementary tables and figure legends**

**Supplementary Figure 1.**

Decision tree model and expression patterns of discriminative proteins for classification of recovered, general PASC, and psychiatric PASC.

**Supplementary Table 1.** List of processed proteins derived from DBS. Seventeen proteins identified by one-way ANOVA as significantly different among the three groups (p < 0.05).

**Supplementary Table 2.**

List of differentially expressed proteins. Differentially expressed proteins (DEPs) identified using Student’s t-test (p ≤ 0.05, ratio ≥ 1.3 or ≤ 0.8) across three pairwise comparisons: recovered vs. psychiatric PASC, recovered vs. general PASC, and general vs. psychiatric PASC.
