# Supplementary figures and images for: Discovery of molecular signature of long-term psychiatric sequelae in COVID-19 through proteome profiling of dried blood spots
Source: Transl Psychiatry. 2025 Oct 10;15:389. doi: 10.1038/s41398-025-03590-2 (PMC12514250; doi:10.1038/s41398-025-03590-2)

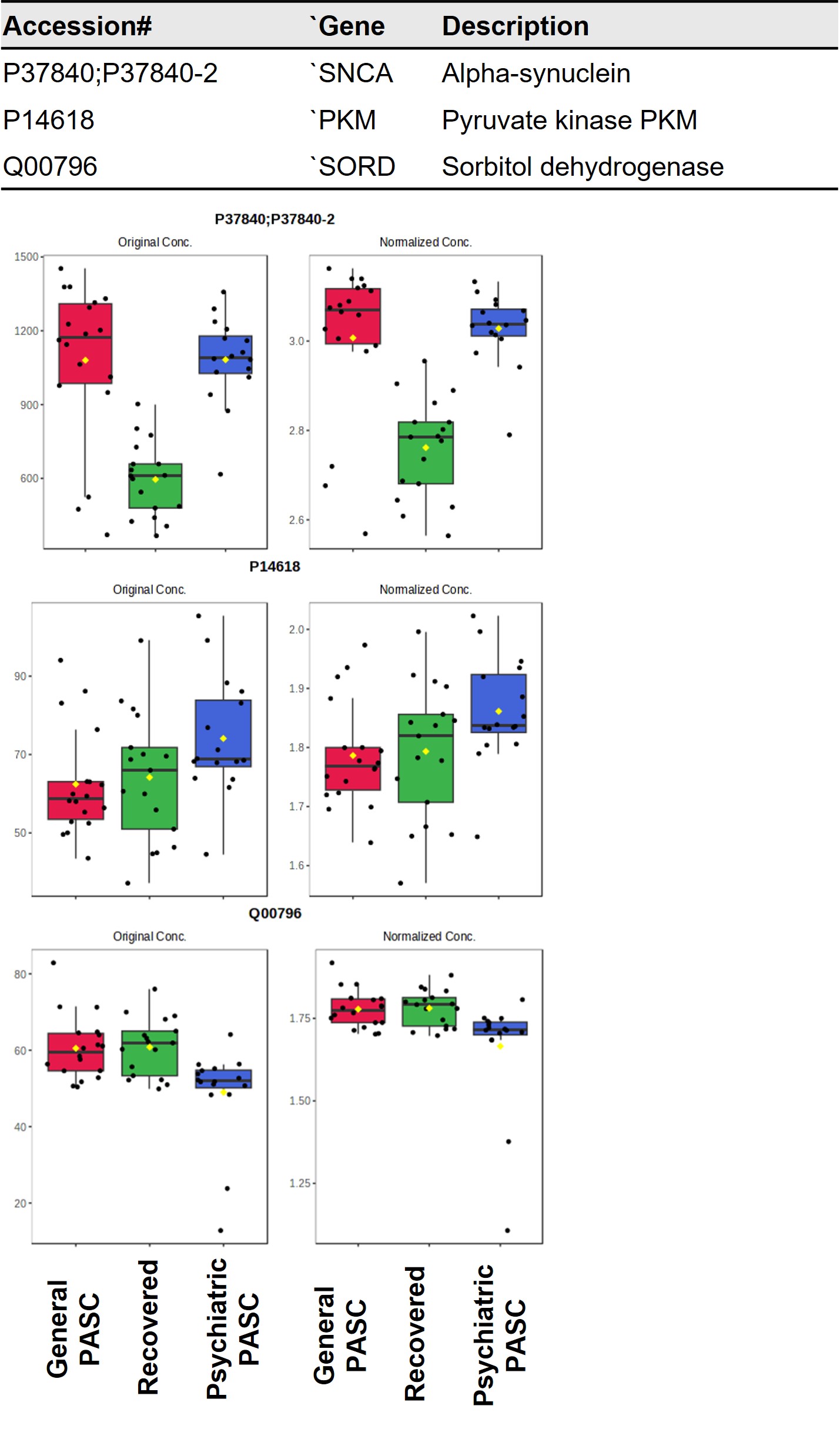

Supplement: Supplementary file 2 — expression level of three proteins [file 41398_2025_3590_MOESM2_ESM.jpg]
